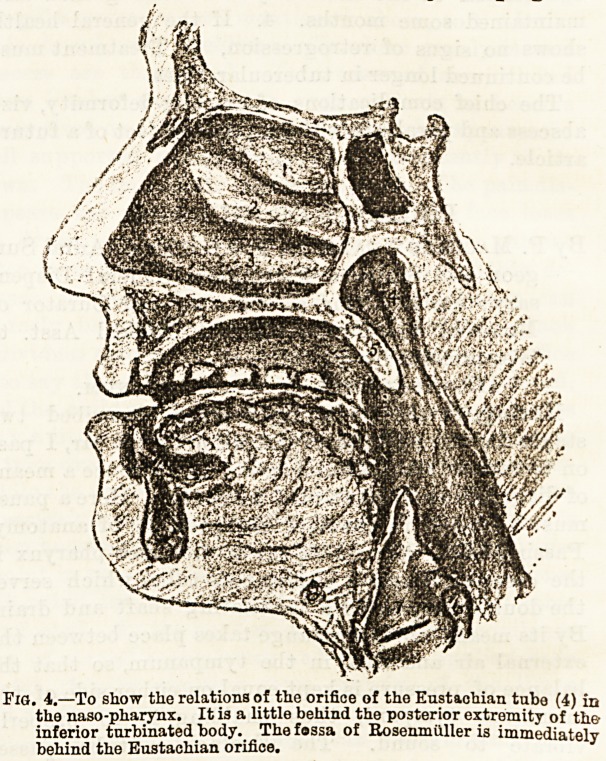# Diseases of the Ear

**Published:** 1894-07-14

**Authors:** P. Macleod Yearsley

**Affiliations:** Aural Surgeon and Surgeon, Farringdon General Dispensary; Assist. Demr. of Anatomy and Curator of Museum, and formerly Aural Clinical Asst. to Westminster Hospital


					DISEASES OF THE EAR.
III.?Inflation of the Tympanum.
By P. Macleod Yearsley, F.R.C.S.Eng., Aural Sur-
geon and Surgeon, Farringdon General Dispen-
sary; Assist. Demr. of Anatomy and Curator of
Museum, and formerly Aural Clinical Asst. to
Westminster Hospital.
Having, in the preceding articles, described two
stages in the physical examination of the ear, I pass
on to certain manipulations which are at once a means
o? diagnosis and a method of treatment. Here a pause
must be made to consider the question of anatomy.
Passing from the middle ear to the naso-pharynx is
the osseo-cartilagious Eustachian tube, which serves
the double purpose of a ventilating shaft and drain.
By its means an interchange takes place between the
external air and that in the tympanum, so that the
balance of pressure is kept equal on either side of the
membrana tympani, thus enabling it to properly
vibrate to sound. The Eustachian tube passes
obliquely downwards and inwards from the tympanum
to the pharynx, its tympanic orifice being 2*5 cm.
higher than the pharyngeal; its length is about 34 to
36 mm., the cartilaginous portion forming two-thirds
of the whole. Its walls being in contact while at rest}
the communication between the ear and the pharynx
is not continuously established, but, two of the palatal
muscles (tensor and levator palati) having some
attachment to the pharyngeal orifice, the tube is
opened by the act of swallowing, a fact utilized in the
procedures about to be described. At the junction
of the osseous and cartilaginous portions (isthmus tubve)
the tube is considerably narrowed, and at that point
strictures most frequently occur. Owing to this con-
tinuity between the pharynx and the ear, morbid con-
ditions of the former are prone to produce effects in
the latter, and the Eustachian catarrh set up by
repeated attacks of pharyngeal inflammation easily
causes obstruction, the proper ventilation of the tym-
panum being thus interfered with and the vibrations
of the membrane disturbed, with consequent impair-
ment of hearing.
In inflation of the tympanum we have not only a
valuable therapeutic agent, but a fairly reliable help
to the diagnosis of the presence of products of disease
in the tympanum, the state of the membrana tyrn-
pani as to perforations, the permeability of the Eus-
tachian tube, and other conditions.
The effect of inflation on the tympanum is a com-
pound one. The Eustachian tube is mechanically
dilated, any secretion it contains is forced into the
pharynx or tympanum (according to whichever end of
^he tube such secretion is situated), and the membrana
tympani is bulged forwards, which movement is com-
municated to the ossicular chain, so that if the drum-
head was previously indrawn, it and the ossicles are
restored to their normal places, and any pressure on
the fluid in the labyrinth removed (thus relieving
tinnitus, to which reference will be made in a future
article), a result helped by the stretching of the
membrane covering the fenestra rotunda. Should
the tympanum contain any products of disease, they
may be partially removed1, but the beneficial results
are chiefly due to the removal of abnormal tension.
For the artificial inflation of the middle ear we have
at our disposal three methods, of which my limited
space will allow but a short description. These
methods are: Yalsalva's experiment, Politzer's douche,
and the Eustachian catheter. The first of these con-
sists inclosing the nostrils and making a strong ex-
piratory effort with the mouth closed, which, by con-
densing the air in the naso-pharynx forces some of it
into the tympanum. The effect, which the surgeon
can hear by means of the diagnostic tube (Fig. 1),
varies with the age, sex, and expiratory strength of the
individual. The method is not very reliable, some-
times failing in normal ears, but, on the other hand,
may succeed where the catheter and Politzerisation
fail. It should, however, always be used, for, although
its value is limited, when it gives a positive result, the
inference is that the Eustachian obstruction is slight,
a fact of some importance in diagnosis.
The second method, which bears Politzer's name,
and was introduced by him in 1868, consists in inflating
the tympanum by means of a rubber balloon, the
nozzle of which is passed into the nostril. Of the appa-
ratus used there are several modifications, that of
Gardiner Brown (Fig. 3) being decidedly the best3.
The mode of application is as follows: The patient
fills his mouth with water, and the nozzle of the douche
is inserted into the nostril and held there by the
surgeon's left hand, the fingers of which gently com-
press the nose ; the patient is then told to swallow,
that they are not so supporting. Over the mammae,
upper part of the chest and crests of the ilia, and
lower part of the abdomen they are necessarily left
unstiffened, and hence allow the body to " settle"
somewhat. These difficulties are to a certain extent
overcome by strengthening them posteriorly with stout
steel bands.
When may treatment be dispensed with in spinal
caries ,J 1. The absence of pain is no test, since this
occurs naturally, if a support be worn ; but if pain ensue
on the removal of the jacket, it must be again resorted
to. 2. When the spine is firmly fixed, and tie de-
formity has remained stationary for some time. 3. If
a recession of the deformity has been gained and
maintained some months. 4. If the general health
shows no signs of retrogression, 5. Treatment must
be continued longer in tubercular cases.
The chief complications of angular deformity, viz.,
abscess and paralysis, will form the subject of a future
article.
DISEASES OF THE EAR.
By P. Macleod Yearsley, F.R.C.S.Eng., Aural Sur-
geon and Surgeon, Farringdon General Dispen-
sary; Assist. Demr. of Anatomy and Curator of
Museum, and formerly Aural Clinical Asst. to
Westminster Hospital.
III.?Inflation op the Tympanum.
Having, in the preceding articles, described two
stages in the physical examination of the ear, I pass
on to certain manipulations which are at once a means
of diagnosis and a method of treatment. Here a pause
must be made to consider the question of anatomy.
Passing from the middle ear to the naso-pharynx is
the osseo-cartilagious Eustachian tube, which serves
the double purpose of a ventilating shaft and drain.
By its means an interchange takes place between the
external air and that in the tympanum, so that the
balance of pressure is kept equal on either side of the
membrana tympani, thus enabling it to properly
vibrate to sound. The Eustachian tube passes
obliquely downwards and inwards from the tympanum
to the pharynx, its tympanic orifice being 2*5 cm.
higher than the pharyngeal; its length is about 34 to
36 mm., the cartilaginous portion forming two-thirds
of the whole. Its walls being in contact while at rest,
the communication between the ear and the pharynx
is not continuously established, but, two of the palatal
muscles (tensor and levator palati) having some
attachment to the pharyngeal orifice, the tube is
opened by the act of swallowing, a fact utilized in the
procedures about to be described. At the junction
of the osseous and cartilaginous portions (isthmus tubx)
the tube is considerably narrowed, and at that point
strictures most frequently occur. Owing to this con-
tinuity between the pharynx and the ear, morbid con-
ditions of the former are prone to produce effects in
the latter, and the Eustachian catarrh set up by
repeated attacks of pharyngeal inflammation easily
causes obstruction, the proper ventilation of the tym-
panum being thus interfered with and the vibrations
of the membrane disturbed, with consequent impair-
ment of hearing.
In inflation of the tympanum we have not only a
valuable therapeutic agent, but a fairly reliable help
to the diagnosis of the presence of products of disease
in the tympanum, the state of the membrana tym-
pani as to perforations, the permeability of the Eus-
tachian tube, and other conditions.
The effect of inflation on the tympanum is a com-
pound one. The Eustachian tube is mechanically
dilated, any secretion it contains is forced into the
pharynx or tympanum (according to whichever end of
the tube such secretion is situated), and the membrana
tympani is bulged forwards, which movement is com-
municated to the ossicular chain, so that if the drum-
head was previously indrawn, it and the ossicles are
restored to their normal places, and any pressure on
the fluid in the labyrinth removed (thus relieving
tinnitus, to which reference will be made in a future
article), a result helped by the stretching of the
membrane covering the fenestra rotunda. Should
the tympanum contain any products of disease, they
may be partially removed1, but the beneficial results
are chiefly due to the removal of abnormal tension.
For the artificial inflation of the middle ear we have
at our disposal three methods, of which my limited
space will allow but a short description. These
methods are : Valsalva's experiment, Politzer's douche,
and the Eustachian catheter. The first of these con-
sists inclosing the nostrils and making a strong ex-
piratory effort with the mouth closed, which, by con-
densing the air in the naso-pharynx forces some of it
Fig. l.
into the tympanum. The effect, which the surgeon
can hear by means of the diagnostic tube (Fig. 1),
varies with the age, sex, and expiratory strength of the
individual. The method is not very reliable, some-
times failing in normal ears, but, on the other hand,
may succeed where the catheter and Politzerisation
fail. It should, however, always be used, for, although
its value is limited, when it gives a positive result, the
inference is that the Eustachian obstruction is slight,
a fact of some importance in diagnosis.
The second method, which, bears Politzer's name,
and was introduced by him in 1868, consists in inflating
the tympanum by means of a rubber balloon, the
nozzle of which is passed into the nostril. Of the appa-
, Pro. 2.
rat us used there are several modifications, that of
Gardiner Brown (Fig. 3) being decidedly the best2.
The mode of application is as follows: The patient
fills his mouth with water, and the nozzle of the douche
is inserted into the nostril and held there by the
surgeon's left hand, the fingers of which gently com-
press the nose ; the patient is then told to swallow,
320 THE HOSPITAL.
July 14, 1894.
and, while doing so, the balloon is firmly and smartly
compressed. Some modification is required with
children, who cannot always be made to understand
when to swallow; the pronunciation of some short
guttural, such as liucTi, generally suffices; the air will,
moreover, enter freely when the child is crying.
The strength of the inflation should depend upon
the amount of obstruction present, and if at first the
results are not very encouraging the surgeon must not
be discouraged, hut should he prepared to persevere.
Inold-standingcases considerable patience is sometimes
required before the air can be made to enter the tympanum
freely. Occasionally the closing of one ear with the
finger will be sufficient to cause the air to enter the
opposite one, but in a large number of cases the
passage of the catheter will be required before the
douche can be used successfully.
Not only is Politzer's method invaluable in case3 of
Eustachian obstruction, but it is a useful adjunct
in the treatment of purulent otitis with perforation, its
employment clearing the tympanum of pus and debris
very efficiently.
When Politzer's douche is not sufficient to inflate
the middle ear, Valsalva's experiment (which, as has
been already said, is of limited value), will nearly
always be useless, and in such cases, or when intra-
tympanic injection of fluids is required,the Eustachian
catheter will have to be used. This instrument (Fig.
2) is made in metal or vulcanite, and it will be found
that the latter substance gives less pain and is better
tolerated by patients. The curve, usually one of 145
degrees, can be altered by immersion in hot water.
Those with an oval opening to the beak are the best,
as they fit the Eustachian tube better.
The catheter is passed thus : Holding it like a pen,
with the beak downwards, the surgeon passes it quickly
along the floor of the nose until he feels it impinge
upon the posterior pharyngeal wall; he then gives it a
half turn outwards, ao that; the beak is directed out-
wards and slightly upwards (the indicating ring being
in a line with the outer canthus of the eye) into the
fossa of Rosenmiiller (Fig. 4) behind the Eustachian
orifice. If the cathether be then gently pulled for-
wards towards the surgeon its point can be felt to slip
over the prominent Eustachian cushion into the open-
ing of the tube, into which it can be pushed.
I am convinced by experience that this method is
the easiest, best, and least likely to fail.
Inflation is carried out by means of a rubber balloon,
the nozzle of which fits into the outer wide end of the
catheter, into which can be placed any medicated fluid
to be injected.
Before closing it is well to mention the absolute
necessity of avoiding inoculation by keeping the
catheter thoroughly aseptic; indeed, it is best to keep a
separate instrument for each patient, and after use it
should be syringed out with and kept in some anti-
septic for twenty-four hours.
1 This is facilitated by inclining the patient's hp-irl  ,j ?,
ways during the inflation. ? In this instm^eK^0^8 "of
rubber and is inflated when the balloon in i ?
plugging the nose without pain to the patient. effectually
Fig. 4.?To show the relations of the orifice of the Eustachian tube (4) in
the naso-pharynx. It is a little behind the posterior extremity of the-
inferior turbinated body. The fossa of Rosenmiiller is immediately
behind the Eustachian orifice.

				

## Figures and Tables

**Fig. 1. f1:**
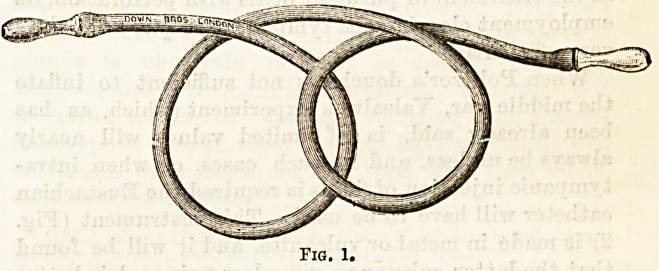


**Fig. 2. f2:**
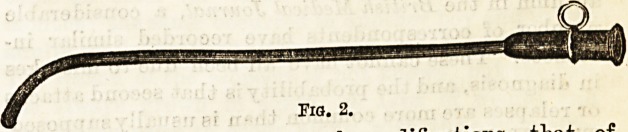


**Fig. 3. f3:**
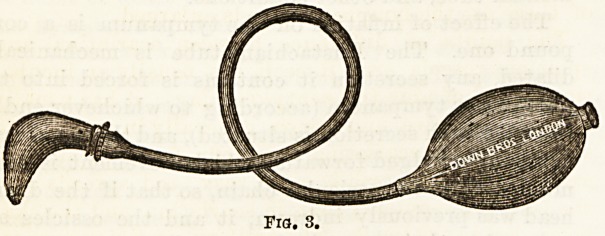


**Fig. 4. f4:**